# HDAC6 inhibitor ACY-1215 enhances STAT1 acetylation to block PD-L1 for colorectal cancer immunotherapy

**DOI:** 10.1007/s00262-023-03624-y

**Published:** 2024-01-17

**Authors:** Yuqing Wen, Shuyu Ye, Zhengshuo Li, Xiaoyue Zhang, Can Liu, Yangge Wu, Run Zheng, Chenxiao Xu, Junrui Tian, Lanjun Shu, Qun Yan, Feiyan Ai, Jian Ma

**Affiliations:** 1 Department of Gastroenterology, The Third Xiangya Hospital, Central South University, Changsha, Hunan China; 2 Cancer Research Institute, School of Basic Medical Science, Central South University, Changsha, Hunan China; 3 NHC Key Laboratory of Carcinogenesis, Key Laboratory of Carcinogenesis and Cancer Invasion of the Chinese Ministry of Education, Hunan Key Laboratory of Nonresolving Inflammation and Cancer, Hunan Key Laboratory of Cancer Metabolism, Changsha, Hunan China; 4 Department of Clinical Laboratory, Xiangya Hospital, Central South University, Changsha, Hunan China

**Keywords:** ACY-1215, HDAC6, PD-L1, STAT1 acetylation, Colorectal cancer immunotherapy

## Abstract

The search for effective combination therapy with immune checkpoint inhibitors (ICI) has become important for cancer patients who do not respond to the ICI well. Histone deacetylases (HDACs) inhibitors have attracted wide attention as anti-tumor agents. ACY-1215 is a selective inhibitor of HDAC6, which can inhibit the growth of a variety of tumor. We previously revealed that HDAC family is highly expressed in colorectal cancer specimens and mouse models. In this study, ACY-1215 was combined with anti-PD1 to treat tumor-bearing mice associated with colorectal cancer. ACY-1215 combined with anti-PD1 effectively inhibited the colorectal tumor growth. The expression of PD-L1 in tumor of mice were inhibited by ACY-1215 and anti-PD1 combination treatment, whereas some biomarkers reflecting T cell activation were upregulated. In a co-culture system of T cells and tumor cells, ACY-1215 helped T cells to kill tumor cells. Mechanically, HDAC6 enhanced the acetylation of STAT1 and inhibited the phosphorylation of STAT1, thus preventing STAT1 from entering the nucleus to activate PD-L1 transcription. This study reveals a novel regulatory mechanism of HDAC6 on non-histone substrates, especially on protein acetylation. HDAC6 inhibitors may be of great significance in tumor immunotherapy and related combination strategies.

**Supplementary Information:**

The online version contains supplementary material available at 10.1007/s00262-023-03624-y.

## Introduction

Colorectal cancer is one of the most common malignant tumors [1]. So far, surgical treatment is still the most important treatment for colorectal cancer patients without distant metastasis [2]. In recent years, targeted therapy and immunotherapy have become the main treatment methods for patients with metastatic colorectal cancer [3].

The immune checkpoint inhibitors have become one of the most widely studied immunotherapies and are increasingly used in cancer patients [4, 5]. Although sustained clinical responses have been observed with the immune checkpoint inhibitors in some patients, most patients still fail to respond to the immunotherapy [6]. In addition, some patients continue to develop resistance after an initial response to the immune checkpoint inhibitors. Therefore, it is of great significance for patients to seek effective combined treatment strategies with immune checkpoint inhibitors [7–9].

We previously revealed that Histone deacetylase (HDAC) family is highly expressed in colorectal cancer specimens and mouse models [10, 11]. Histone deacetylase 6 (HDAC6) is a unique class of IIb HDAC, mainly localized in the cytoplasm. HDAC6 not only modifies and regulates histones, but also acts on some non-histone [12]. HDAC6 is involved in the regulation of tumor cell proliferation, apoptosis, migration and invasion, autophagy, DNA damage response and immune regulation [13–15]. As a result, HDAC6 can be considered as a tumor target. ACY-1215 is a selective HDAC6 inhibitor currently in phase I/ II clinical trials [16]. In recent years, studies have reported that ACY-1215 has antitumor effects in a variety of solid tumors [17–19]. PD-L1 expressed on the surface of tumor cell membrane is a common biomarker for tumor therapy and is closely related to the clinical response of anti-PD1/PD-L1 [20]. Previous studies have also reported that HDAC6 regulates the expression of PD-L1 [21, 22]. In addition, ACY-1215 plays a regulatory role in immune response [23, 24]. However, the specific regulatory mechanism of HDAC6 on PD-L1 is not clear, and whether ACY-1215 can improve the therapeutic effect of anti-PD1/PD-L1 and its function in anti-tumor immunity need to be further clarified.

This study sought to investigate the antitumor efficacy of ACY-1215 in combination with anti-PD1, and the role and mechanism of ACY-1215 in the immunotherapy of colorectal cancer. Herein, the study reveals a new mechanism by which HDAC6 down-regulates PD-L1 by regulating non-histone substrates, and provides a new strategy for the tumor immunotherapy in colorectal cancer.

## Materials and methods

### Cell culture

The mouse colon cancer cell lines MC38 and CT26, the human colorectal cancer cell lines HCT116 and SW480 were cultured in RPMI-1640 medium (Biological Industries, Cat No. 01-100-1 A, Israel) supplemented with 10% fetal bovine serum (Tsingmu Biotechnology, Cat No. mu001SR, China). Test for *Mycoplasma* contamination was performed before or after experiments. They were verified to be negative.

### Clinical tissue samples

The tumor tissues and adjacent normal tissue samples of colorectal cancer patients randomly collected from Xiangya Hospital were prepared into paraffin sections for H&E staining pathological analysis and immunohistochemical detection of HDAC6. Written informed consent was obtained from all study participants. The collection and use of tissue samples were approved by the Ethical Review Committee of the Xiangya Hospital of Central South University.

### Animal models

C57BL/6 female mice aged 5–6 weeks were purchased from Hunan SJA Laboratory Animal Co., Ltd. MC38 cells (1 × 10^6^) were inoculated subcutaneously into the right shoulder of mice. On day 0, C57BL/6 mice inoculated with the same amount of MC38 cells were randomly divided into 4 groups: (a) injected with sterile PBS (as the negative control); (b) only HDAC6 inhibitor (ACY-1215) (50 mg/kg) monotherapy; (c) only anti-PD1 (5 mg/kg) monotherapy; (d) ACY-1215 (50 mg/kg) combined with anti-PD1 (5 mg/kg). When the tumor volume of the tumor-carrying mice reached about 100 mm^3^ (day 9 after MC38 cells inoculation), the mice were treated by intraperitoneal injection with ACY-1215, anti-PD1 or PBS according to the group. The drug was administered every two days and the tumor size of the mice was recorded. The protocol was approved by the Animal Ethics Committee of Central South University. ACY-1215 was purchased from Selleck Biotechnology (Cat No. S8001), and the anti-mouse PD-1 antibody (RMP1-14) was purchased from BioXCell (Cat No. BP0146).

### Immunohistochemistry

The paraffin-embedded tissues were cut 4 μm thick, then deparaffinized and rehydrated. The expressions of HDAC6 (1:50; Cat No. ab133493; Abcam), CD8a (1:50; Cat No. A11856; ABclona), Granzyme B (1:2000; Cat No. ab255598; Abcam), and TBX21 (1:100; Cat No.13700-1-AP; Proteintech) were detected by immunohistochemical staining as described previously [25].

### Immunofluorescence

Cells were immersed in the medium containing 3.7% paraformaldehyde and fixed for 1 h, then permeabilized with 0.25%Triton X-100, and blocked with normal goat serum. The primary antibodies were added (different primary antibodies were added according to different experiments: STAT1, p65 or HDAC6) and incubated at room temperature for 2 h. Then, Alexa Fluor 488-conjugated or 568-conjugated secondary antibodies (Beyotime) was added and incubated for 1 h. The stained cells were observed using a fluorescence microscope.

### Quantitative real-time PCR

Total RNA was extracted from tumor tissue or cultured cells with TRIzol according to the standard protocol. cDNA was synthesized from 2 µg total RNA with a Revert Aid RT Reverse Transcription Kit (Thermo Scientific, Waltham, MA). The operation procedure of reverse transcription PCR (RT-PCR) is as follows. The first step is to run at 65 ℃ for 5 min and terminate the program when the temperature drops to 4 ℃. The second step is to run at 42 ℃ for 60 min, then run at 70 ℃ for 5 min, and terminate the program when the temperature drops to 4 ℃. The mRNA level was evaluated using SYBR Green real-time qPCR (Takara). The RT-PCR analysis was performed using a Bio-Rad CFX96 Real-Time System (Bio-Rad, Hercules, CA). Mouse or human *GAPDH* was amplified in parallel as an internal control. Expression of each gene was quantified by measuring cycle threshold values, and the 2^–ΔΔCt^ method was used to calculate relative changes in gene expression. The primers for quantitative PCR (qPCR) are listed in Supplemental Table 1.

### Western blotting

Extracts of cells or tissue lysed with RIPA buffer were cleared by centrifugation. Lysates (30 µg of protein) were subjected to SDS-PAGE, and the separated bands were transferred to a polyvinylidene difluoride membrane (Millipore) that was then probed with various antibodies. The following antibodies were used: anti-PD-L1 (1:1000; Cat No. 66248-1-Ig; Proteintech), anti-IFN-γ (1:200; Cat No. bs-0480R, Bioss), anti-STAT1 (1:2000; Cat No. 10144-2-AP; Proteintech), anti-pSTAT1 (Tyr701) (1:500; Cat No. AF3300, Affinity), anti-Ac-lys (1:500; Cat No. A2391, ABclonal), anti- NF-κB p65 (1:1000; Cat No. ET1603-12, Huabio), anti-HDAC6 (1:1000; Cat No. ab133493, Abcam), anti-Histone H3 (1:1000; Cat No. A2348, ABclonal), and anti-GAPDH (1:1000; Cat No. ET1601-4, Huabio). Cytoplasmic and nuclear fractions were isolated with nuclear and cytoplasmic protein extraction kits (Beyotime).

### Co-immunoprecipitation

Endogenous co-immunoprecipitation (co-IP) was performed as we previously described [26]. Briefly, cell lysates were incubated with 2 µg antibody at 4 °C overnight. Protein A/G-Sepharose beads (Bio-linkedin) were added, and the mix was incubated for 2 h at 4 °C. The immunocomplexes were washed three times with PBST. Bound proteins were eluted by boiling with 2×SDS loading buffer before being resolved by SDS-PAGE.

### T-cell mediated tumor cell killing assay

T cell killing assay was performed as we previously described [27]. Activated T cells were obtained as described previously [28]. Peripheral blood was collected from healthy donors, and peripheral blood mononuclear cells were extracted from the lymphocyte isolation solution (Cat No. LTS1077, TBD). The isolated peripheral blood mononuclear cells were cultured in RPMI-1640 medium supplemented with 10% fetal bovine serum, Immuno Cult Human CD3/CD28/CD2 T cell activator (Cat No.10,970, STEMCELL Technologies) and IL-2 (50 ng/mL, 200-02-50, PeproTech) for 1 week according to the manufacturer’s protocol. Once adhering to the plates, HCT116 or SW480 cells were treated with IFN-γ for 24 h, incubated with ACY-1215 (0, 2.5, 5.0 µM) for 24 h, then processed with activated T cells for 24 h. The ratio between tumor cells and activated T cells is 1:3. T cells and tumor cell debris were removed by PBS buffer, and remaining adherent tumor cells were quantified by cell counting kit-8 or crystal violet staining.

### Flow cytometry

Activated T cells were obtained as described above. Once adhering to the plates, SW480 cells were treated with IFN-γ for 24 h, incubated with ACY-1215 (0, 2.5, 5.0 or 10.0µM) for 24 h, then processed with activated T cells for 24 h. The ratio between tumor cells and activated T cells is 1:8. Before T cells were collected, the tumor cells and T cells were co-cultured for 3 h with the addition of the Golgi transport blocker 25 ng/mL BFA (Selleck). Then T cells were collected and stained with cell membrane antibodies APC/Cvanine7-CD3 (Cat No. 300,317, BioLegend), PE/Cyanine7-CD8a (Cat No. 300,913, BioLegend). After pretreatment with a membrane breaker, the fluorescently labeled cytokine Brilliant Violet 421^TM^-IFN-γ (Cat No. 502,531, BioLegend) and PE-Granzyme B (Cat No. 3,722,071, BioLegend) antibody was added to incubate. In addition, to detect the expression of PD-L1 on the membrane of single-cell suspension from human cell lines in culture, the PE-PD-L1 (Cat No. 329,706, BioLegend) antibody is used. Flow cytometry was performed using the BD FACS Calibur flow cytometer. The FlowJo v10 software (Treestar) was used for data analysis.

### Luciferase reporter assay

The luciferase reporter gene vector was kindly gifted by Prof. Xiong wei from Cancer Research Institute, Central South University, China. HCT116 cells were co-transfected with luciferase reporter vectors constructed with the PD-L1 promoters, pRL-TK Renilla luciferase vector, and STAT1 plasmid carrier (or NF-κB p65 plasmid carrier). Luciferase activity was measured using the Dual-Luciferase Reporter Assay System (Cat No. FR201-01, TransGen Biotech) by plate reader.

### In vitro chemical acetylation and enzymatic deacetylation

Human recombinant STAT1 (Sino Biological Inc. Cat#12,766-H20B) was mixed with acetic anhydride solution of different concentrations (final concentration: 0, 0.2, 0.4, 0.6, 0.8, 1.0, 3.0, 6.0mM) and reacted at room temperature for 120 min. The pH of the reaction system was adjusted to 8 by adding 1 M NaOH. An equal amount of 4% SDS Sample buffer was added to the reaction solution. After boiling the sample at 95 °C for 5 min, the reaction solution was divided into two parts. One part was used for western blot assay to detect STAT1 acetylation level by Acetyl-Lysine antibody. The other part of reaction solution was used to determine the total STAT1 protein by Coomassie blue staining for SDS-PAGE glue.

After determining the appropriate acetic anhydride concentration, the chemical acetylation reaction system of STAT1 was expanded by 10 times, and sufficient STAT1 protein participated in the acetylation reaction. After neutralization with 1 M NaOH, the neutralization solution was centrifugally concentrated using Pierce Protein Concentrator, and 1 mL HDAC6 deacetylation reaction buffer I (50 mM Tris-HCl pH8.0, 137 mM NaCl, 2.7 mM KCl, 1 mM MgCl_2_), repeated 3 times to ensure that the final concentration of acetylated STAT1 was about 1 µg/µL. Appropriate amount of acetylated STAT1 was added to human recombinant HDAC6 (Active Motif, Cat#31,543) and HDAC6 deacetylation buffer II (50 mM Tris-HCl pH8.0, 137 mM NaCl, 2.7 mM KCl, 1 mM MgCl_2_, 1 mg/mL BSA). ACY-1215 (1mM or 2mM) was added to HDAC6 inhibitor group to inhibit the deacetylation activity of HDAC6 in vitro. After reaction at 37 °C for 90 min, the equal amount of 4% SDS Sample buffer was added to the reaction solution. After boiling the sample at 95 °C for 5 min, the reaction solution was divided into two parts. One part was used for western blot assay to detect STAT1 acetylation level by Acetyl-Lysine antibody. The other part of reaction solution was used to determine the total STAT1 protein by Coomassie blue staining for SDS-PAGE glue.

### Statistical analysis

Statistical analyses were performed using SPSS 27 and Prism 8.0. The unpaired t-test was used to evaluate the significant difference between two groups of data. Two-way ANOVA was used to compare mouse tumor volume data among different groups. The results are expressed as the means ± SD. A *p* value < 0.05 was considered statistically significant.

## Results

### HDAC6 is highly expressed in colorectal cancer

It’s previously reported that HDAC family is highly expressed in colorectal cancer specimens and mouse models [10, 11]. To investigate the expression of HDAC6 in colorectal cancer, tumor tissue and adjacent normal tissue samples were collected from patients with colorectal cancer, and immunohistochemistry assay revealed that HDAC6 is expressed at a higher level in the tumor tissues than in the adjacent normal tissues (Fig. S1A). Bioinformatic analysis discovered that HDAC6 was elevated in colorectal cancers compared to the normal tissues in the Cancer Genome Atlas (TCGA) database [29, 30], as well as in the UALCAN online website (http://ualcan.path.uab.edu/) (Fig. S1B,C). Colorectal cancer patients with high HDAC6 expression had worse survival comparing with that of low HDAC6 patients (*p* < 0.05) (Fig. S1D).

### ACY-1215 plays an additive antitumor role with anti-PD1

**Fig. 1:**
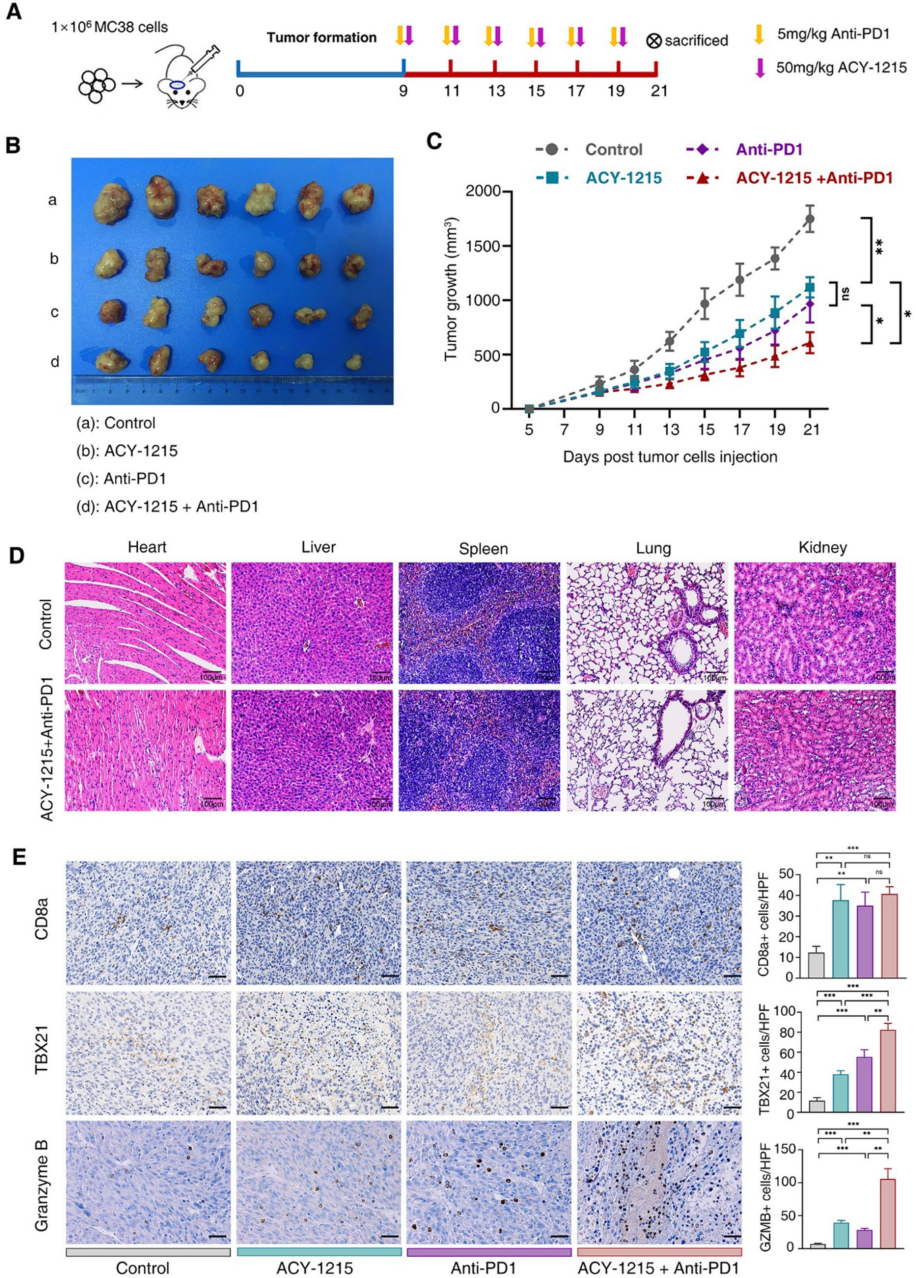
The HDAC6 inhibitor (ACY-1215) plays an additive antitumor effect with anti-PD1. **A** The antitumor effects of ACY-1215 and the immune checkpoint inhibitors (anti-PD1) in vivo were investigated in a mouse transplanted tumor model. Please refer to “Materials and Methods” for specific construction methods of animal models. **B** 21st days after MC38 cells injection, the C57BL/6 mice were sacrificed to observe tumor mass. **C** Tumor growth curve of each group. The tumor volume was measured every two days from day 9 to 21 after MC38 cell inoculation. **D** Photomicrographs illustrating hematoxylin-eosin staining in the heart, liver, spleen, lung, and kidney of the tumorous mice. Scale bar: 100 μm. **E** The expressions of CD8a, TBX21 and Granzyme B in tumor tissues of tumor-bearing mice were assayed by immunohistochemistry and quantitatively analyzed. Scale bar: 50 μm

ACY-1215 is a selective inhibitor of HDAC6, which has good therapeutic effect on hematological malignant tumors and has entered clinical trials [31]. However, the study of ACY-1215 in solid tumors is limited, and its therapeutic effect against solid tumors is unclear. To explore whether ACY-1215 has an antitumor effect on the colorectal cancer and whether it can synergize with the immune checkpoint inhibitor (anti-PD1), the mouse model with subcutaneous colorectal tumor was constructed. When the tumor volume of the tumor-bearing mice reached about 1 × 100 mm^3^, the mice were treated with ACY-1215 and anti-PD1 (or PBS as negative control) by intraperitoneal injection (Fig. 1A). The mice were dosed every two days and the tumor sizes were recorded. On the 21st day, the tumor-bearing mice were sacrificed. Compared with the control group, the tumor growth of mice was inhibited in the ACY-1215 or anti-PD1 monotherapy group and the combination treatment group. The mice in the ACY-1215 combined with anti-PD1 treatment group had the smallest tumor volume and the slowest tumor growth (Fig. 1B, C). The pathological examinations were performed on the heart, liver, spleen, lung and kidney of mice, and the results showed that ACY-1215 and anti-PD1 had no toxic and the side effect on the pathological structure of the visceral organs of mice (Fig. 1D). These suggested that ACY-1215 had a certain antitumor effect in tumor-bearing mice and acts additively with anti-PD1.

Error bars represent SD. *p < 0.05, **p < 0.01, ***p < 0.001, in two-tailed unpaired t test. Each group has six mice. ns, not significant.

### ACY-1215 promotes Ifn-γ expression and down-regulates the expression of Pd-l1 in tumor tissues of tumor-bearing mice

Previous studies have shown that HDAC6 can participate in the immunomodulatory process of tumor occurrence and development by up-regulating the expression of PD-L1 [32, 33]. In order to investigate whether ACY-1215 achieves therapeutic effect in tumor-bearing mice through its role in anti-tumor immunity, the activation of T cells and the expression of Pd-l1 in tumor-bearing mice were analyzed. Tumor tissues of mice were taken and made into pathological sections, and the functional biomarkers that can reflect T cell activation, such as Cd8, Tbx21 and Granzyme B, were detected by immunohistochemistry. Expressions of Cd8, Tbx21 and Granzyme B in tumor tissues of ACY-1215 monotherapy group and anti-PD1 monotherapy group were higher than those of control group. Expression of Cd8 of the ACY-1215 combined with anti-PD1 group was significantly higher than that of control group. Expressions of Tbx21 and Granzyme B of the ACY-1215 combined with anti-PD1 group were significantly higher than those in the control group as well as the monotherapy group (Fig. 1E).

**Fig. 2:**
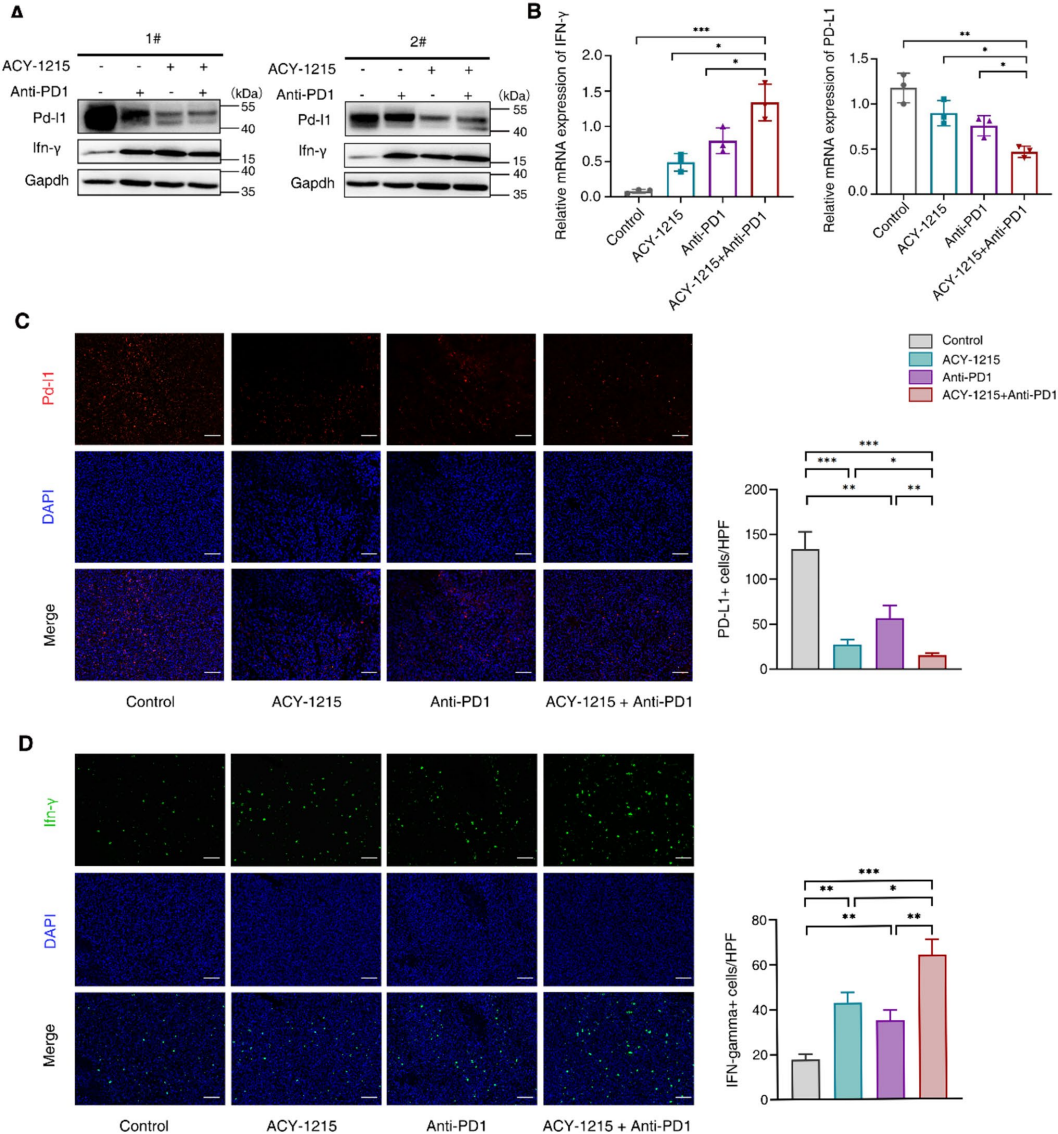
ACY-1215 promotes IFN-γ expression and down-regulates the expression of PD-L1 in tumor tissues of tumor-bearing mice. **A** The total protein was extracted from tumor tissues of tumor-bearing mice in different groups. The expression of Pd-l1 and Ifn-γ in tumor tissues were assayed by Western Blot. **B** The total RNA was extracted from tumor tissues, and the expression of Pd-l1 and Ifn-γ in different tumor tissues were assayed by quantitative real-time PCR. **C** The expression of Pd-l1 in tumor tissues was assayed by immunofluorescence. Pd-l1 is the red fluorescence, the nucleus is stained by DAPI, and “Merge” represents the signal superposition diagram of Pd-l1 and DAPI. Scale bar: 50 μm. **D** The expression of Ifn-γ in tumor tissues was assayed by immunofluorescence. Ifn-γ is the green fluorescence, the nucleus is stained by DAPI, and “Merge” represents the signal superposition diagram of Ifn-γ and DAPI. Scale bar: 50 μm

IFN-γ is a biomarker for reflecting the activation of T cells. The expression levels of Pd-l1 in the ACY-1215 monotherapy group and the ACY-1215 combined with anti-PD1 group were lower than those in the control group, while the expression of Ifn-γ was higher than that of the control group. Among them, the expression of Pd-l1 was the lowest in the ACY-1215 combined with anti-PD1 group, while the expression of Ifn-γ was the highest in the ACY-1215 combined with anti-PD1 group (Fig. 2A,B). In addition, the immunofluorescence was also used to observe the expression of Pd-l1 and Ifn-γ in tumor tissues. The fluorescence intensity (red) of Pd-l1 in tumor tissues of the ACY-1215 monotherapy group and ACY-1215 combined with anti-PD1 group was weaker than that in the control group, and the fluorescence distribution range was smaller than that in the control group (Fig. 2C). The fluorescence intensity (green) of Ifn-γ in the ACY-1215 monotherapy group and ACY-1215 combined with anti-PD1 group was stronger than that of the control group, and the fluorescence distribution range was larger than that of the control group (Fig. 2D). These results indicated that ACY-1215 promoted the activation of T cells in tumor tissues of tumor-bearing mice, possibly through down-regulating Pd-l1.

Error bars represent SD. *p < 0.05, **p < 0.01, ***p < 0.001, in two-tailed unpaired t test.

### ACY-1215 down-regulates the expression of PD-L1 in colorectal cancer cells

**Fig. 3:**
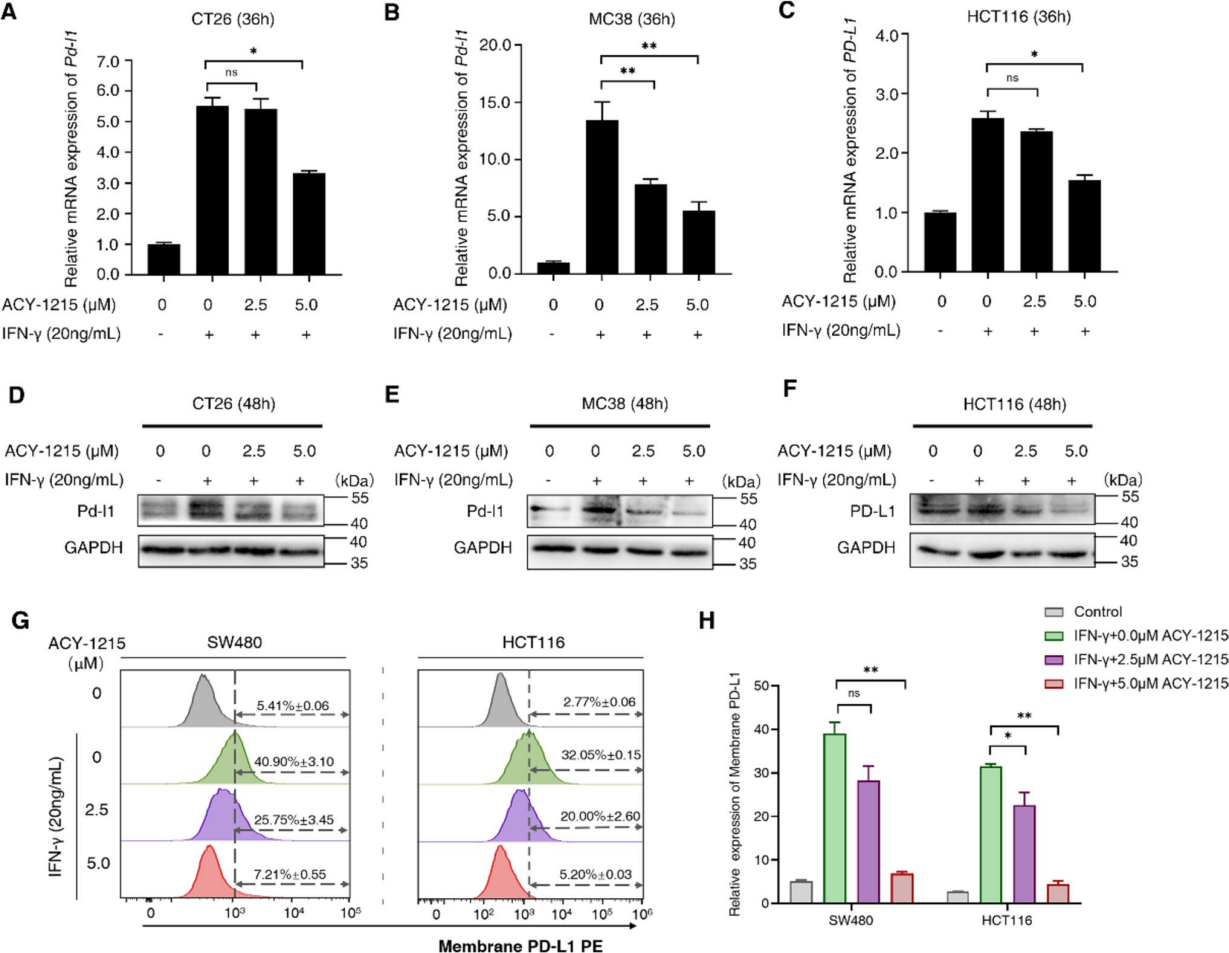
ACY-1215 inhibits the expression of PD-L1 in colorectal cancer cells  CT26, MC38, and HCT116 cells were pretreated with 20 ng/mL IFN-γ, followed by increased concentrations of ACY-1215 (0, 2.5, 5.0 µM). **A**–**C** The total RNA was extracted from cells after ACY-1215 treatment for 36 h, and the expression of PD-L1 was assayed by quantitative real-time PCR. **D**–**F** The total proteins were extracted after ACY-1215 treatment for 48 h, and the expression of PD-L1 protein was assayed by Western Blot. **G**–**H** SW480 and HCT116 cells were pretreated with 20 ng/mL IFN-γ, and then treated with the increased concentrations (0, 2.5, 5.0 µM) of ACY-1215 for 24 h. The expression of PD-L1 on tumor cell membrane was assayed by flow cytometry, and quantitative statistics were performed. Three independent repeated experiments were performed. Error bars represent SD. *p < 0.05, **p < 0.01, ***p < 0.001, in two-tailed unpaired t test

Next, whether ACY-1215 regulates the expression of PD-L1 in colorectal cancer cell lines (CT26, MC38, HCT116 and SW480) was investigated. Cells were pretreated with IFN-γ for 24 h to enhance the expression of PD-L1, and then treated with ACY-1215 (0, 2.5, 5.0 µM). IFN-γ treatment significantly increased the expression of PD-L1, whereas ACY-1215 inhibited the expression of PD-L1 in a dose-dependent manner (Fig. 3). This suggested that the HDAC6 inhibitor, ACY-1215, suppressed the expression of PD-L1 in colorectal cancer cells.

### ACY-1215 promotes the killing effect of T cells on tumor cells in vitro

**Fig. 4:**
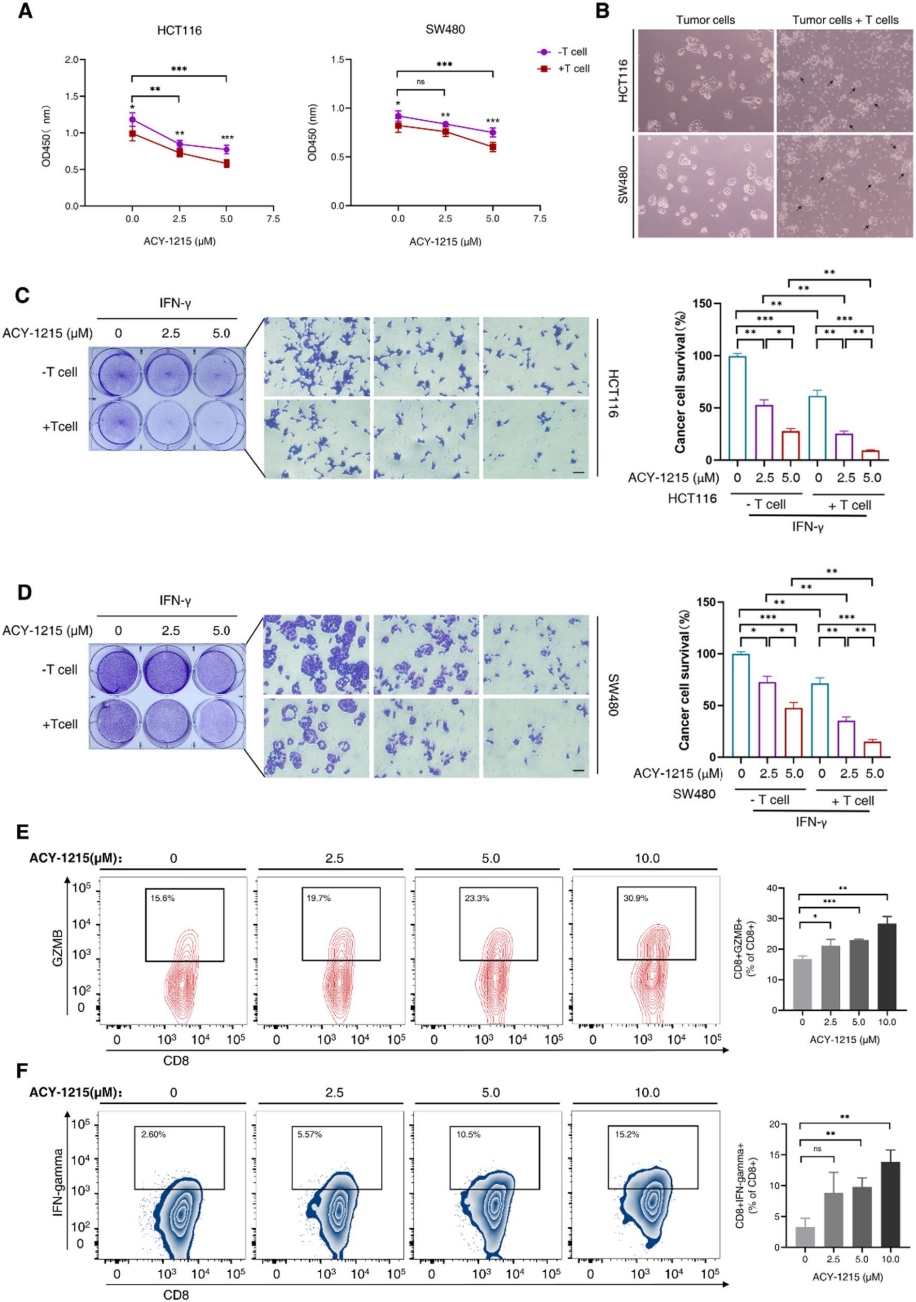
ACY-1215 promotes the killing effect of T cells on tumor cells in vitro. **A**–**D** The colorectal cancer cells HCT116 and SW480 were used for co-culture experiments of T cells and tumor cells. Once adhering to the plates, HCT116 and SW480 cells were treated with IFN-γ for 24 h, incubated with ACY-1215 (0, 2.5, 5.0 µM) for 24 h, then processed with activated T cells for 24 h. The ratio of tumor cells to T cells was 1:3. After coculture, the fragments of T cells and tumor cells were washed away with PBS buffer, and the rest of the tumor cells were detected with cell counting kit-8 (**A**) or crystal violet staining (**C** and **D**), and quantitative analysis was performed. Error bars represent SD. *p < 0.05, **p < 0.01, ***p < 0.001, in two-tailed unpaired t test. Scale bar: 100 μm. (**B**) The morphology of cells before T cells are treated with tumor cells and when T cells are co-cultured with tumor cells. **E** and **F** Once adhering to the plates, SW480 cells were treated with IFN-γ for 24 h, incubated with ACY-1215 (0, 2.5, 5.0 or 10.0 µM) for 24 h, then processed with activated T cells for 24 h. The ratio of SW480 cells to T cells was 1:8. Before T cells were collected, the tumor cells and T cells were co-cultured for 3 h with the addition of the Golgi transport blocker 25 ng/mL BFA. After treatment, T cells suspended in the medium in the co-culture system were collected as far as possible. The effects of ACY-1215 (0, 2.5, 5.0, 10.0 µM) on the secretion of granzyme B and IFN-γ by cytotoxic T cells were assayed by flow cytometry. Three independent repeated experiments were performed. Error bars represent SD. *p < 0.05, **p < 0.01, ***p < 0.001, in two-tailed unpaired t test

PD-L1 of tumor cells binds to PD-1 on the surface of T cells to inhibit the proliferation and function of T cells, through which the tumor cells evade the immune surveillance. In order to explore whether ACY-1215 affects the killing effect of T cells on tumor cells by suppressing PD-L1, HCT116 and SW480 cells were selected for in vitro co-culture experiments of T cells and tumor cells. After co-culture, the fragments of T cells and tumor cells were washed away with PBS buffer solution, and the remaining adherent tumor cells were assayed by cell counting kit (Fig. 4A). The morphology changes of cancer cells before or after co-culturation were observed (Fig. 4B). In the treatment group with the addition of ACY-1215 and T cells, the killing effect of T cells on tumor cells was enhanced in a concentration-dependent way as the concentration of ACY-1215 increased (Fig. 4A, C and D).

The proliferation, apoptosis or ability to secrete effector cytokines (e.g. Granzyme B and IFN-γ) of T cells are related to their killing effect on tumor cells. Total T cell activity (CD3^+^) and cytotoxic T cells (CD8^+^) activity were almost unaffected by increased concentration of ACY-1215 after co-culture for 24 h (Fig. S2), while the cytotoxic T cells secreting Granzyme B (CD8^+^GZMB^+^) and the cytotoxic T cells secreting IFN-γ (CD8^+^IFN-γ^+^) showed a concentration-dependent increase upon ACY-1215 treatment (Fig. 4E,F). These results indicated that ACY-1215 improved the ability of cytotoxic T cells to secrete granzyme B and IFN-γ in the co-culture system, thereby promoting the killing effect of cytotoxic T cells on tumor cells in vitro.

### ACY-1215 down-regulates PD-L1 by promoting STAT1 acetylation

**Fig. 5:**
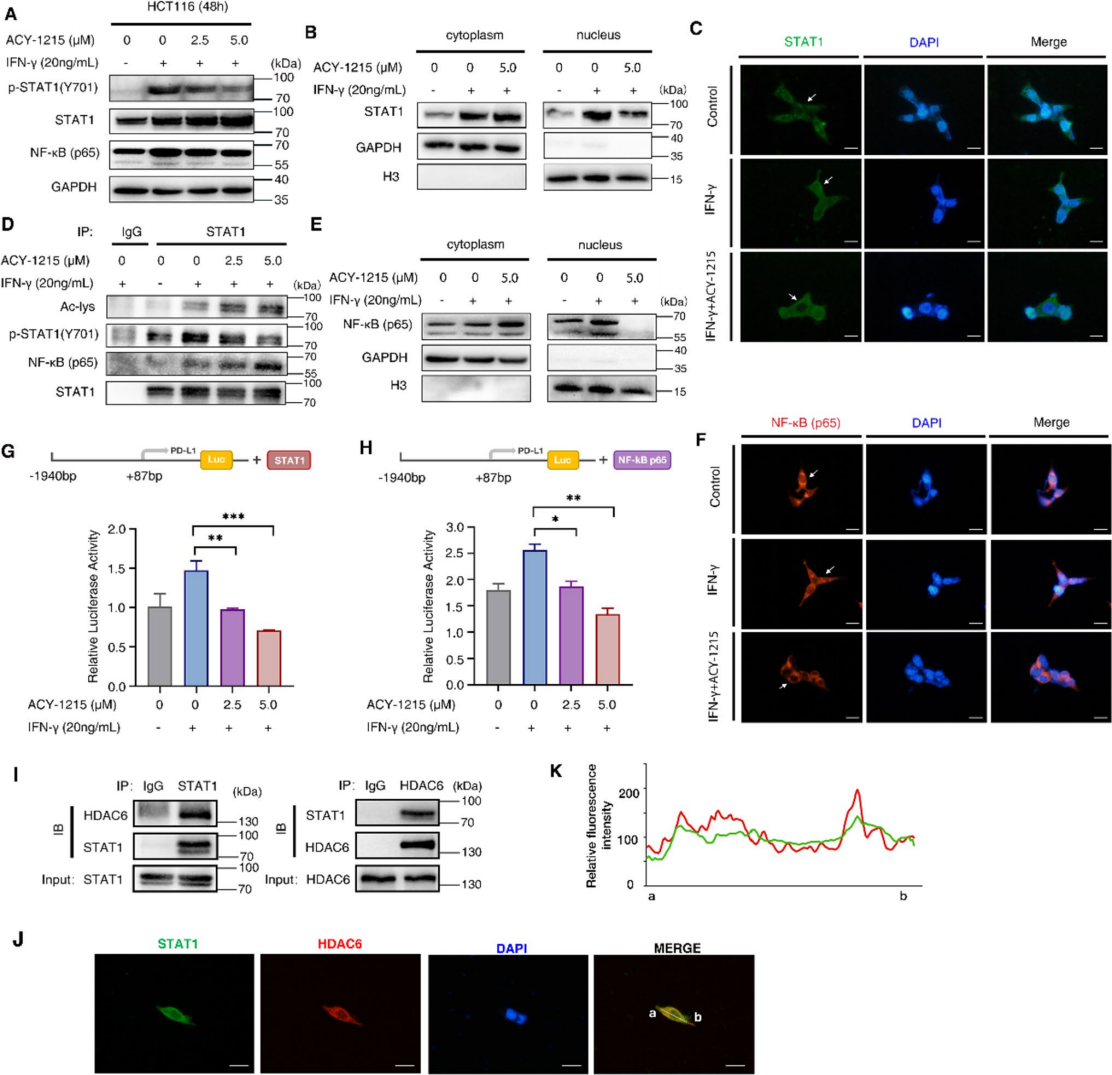
ACY-1215 down-regulates PD-L1 by promoting STAT1 acetylation.  HCT116 cells were pretreated with 20 ng/mL IFN-γ, and then treated with ACY-1215 (0, 2.5, 5.0 µM) for 48 h to extract the total protein of the cells for Western blot experiments, or treated with ACY-1215 (0, 5.0 µM) for 24 h in immunofluorescence experiments. **A**, **B** The expression of phosphorylated STAT1 (p-STAT1) and NF-κB p65 in whole cells were detected by Western Blot (**A**); the expression of STAT1 proteins in cytoplasm and nucleus was detected by Western blot (**B**). GAPDH is the cytoplasmic reference protein and H3 is the nuclear reference protein. **C** The effect of ACY-1215 on STAT1 entry into the nucleus was detected by immunofluorescence. STAT1 was the green fluorescence, the nucleus was stained by DAPI, “Merge” represents the signal superposition diagram of STAT1 and DAPI. Scale bar: 50 μm. **D** The effects of ACY-1215 on IFN-γ-induced STAT1 phosphorylation and acetylation were determined by immunoprecipitation. **E** The expression of NF-κB p65 in cytoplasm and nucleus was detected by Western blot. GAPDH is the cytoplasmic reference protein and H3 is the nuclear reference protein. **F** The effect of ACY-1215 on NF-κB p65 entry into the nucleus was detected by immunofluorescence. NF-κB p65 was the red fluorescence, the nucleus was stained by DAPI, “Merge” represents the signal superposition diagram of NF-κB p65 and DAPI. Scale bar: 50 μm. **G** and **H** Once adhering to the plates, HCT116 cells were co-transfected with luciferase reporter vectors constructed with the PD-L1 promoters, pRL-TK Renilla luciferase vector, STAT1 plasmid vector (**G**) or NF-κB p65 plasmid vector (**H**). After transfection for 24 h, the transfected HCT116 cells were pretreated with 20 ng/mL IFN-γ, and then treated with ACY-1215 for 24 h. The cell lysates were collected and the activity of luciferase was detected. **I** The endogenous interaction between STAT1 and HDAC6 in HCT116 cells was analyzed by co-immunoprecipitation. **J** The co-localization of STAT1 and HDAC6 in HCT116 cells was detected by immunofluorescence. Scale bar: 50 μm. **K** The fluorescence signal distribution of STAT1 and HDAC6 was measured by ImageJ software. Three independent repeated experiments were performed. Error bars represent SD. *p < 0.05, **p < 0.01, ***p < 0.001, in two-tailed unpaired t test

IFN-γ mainly activates the JAK/STAT1 signaling to regulate the transcription of hundreds of genes including PD-L1. To further investigate the mechanism of PD-L1 down-regulation, the effect of ACY-1215 on STAT1 signaling was investigated. IFN-γ alone increased the expression level of phosphorylated STAT1 (p-STAT1). After treatment with ACY-1215, the expression level of p-STAT1 decreased in a concentration-dependent manner as the concentration of ACY-1215 increased (Fig. 5A). In addition, ACY-1215 decreased the expression of STAT1 in nucleus (Fig. 5B,C).

HDAC6 is a histone deacetylase that regulates the acetylation of its substrates. STAT1 acetylation can inhibit IFN-α-induced STAT1 phosphorylation, nuclear translocation and the transcription of downstream target genes [34]. It was crucial to find out whether the inhibition of STAT1 phosphorylation and nuclear translocation by ACY-1215 is related to its regulation of STAT1 acetylation. Upon ACY-1215 treatment, the acetylation level of STAT1 increased, while the phosphorylation level of STAT1 decreased (Fig. 5D). In addition, the up-regulation of STAT1 acetylation also increased STAT1-bound NF-κB p65 (Fig. 5D), while the total NF-κB p65 in whole cells did not change with ACY-1215 treatment (Fig. 5A). This suggested that ACY-1215 may retain NF-κB p65 in the cytoplasm by inhibiting the entry of STAT1 into the nucleus. To verify the hypothesis, the cytoplasmic and nuclear proteins of HCT116 cells were extracted, and the western blot assay showed that ACY-1215 inhibited the expression of NF-κB p65 in the nucleus (Fig. 5E). Immunofluorescence assay also showed that the expression of NF-κB p65 in the nucleus was enhanced under IFN-γ treatment, whereas it was significantly decreased when ACY-1215 was added (Fig. 5F).

To verify whether HDAC6 is the deacetylase of STAT1, we designed an experiment to chemically acetylate the full-length human STAT1 protein (hSTAT1) with acetic anhydride and incubate it with human recombinant HDAC6 (hHDAC6) after purification to detect changes in the degree of acetylation (Fig. S3) [35, 36]. With the increase of acetic anhydride concentration, the acetylation level of STAT1 protein gradually increased and was relatively highest when the acetic anhydride concentration was 6 mM (Fig. S3A). We used 6 mM acetic anhydride for chemical acetylation reaction, the reaction products were concentrated and replaced into the deacetylation reaction buffer, and the purified acetylated STAT1 was then deacetylated with human HDAC6 protein in vitro (Fig. S3B). Western blot results showed that in the presence of HDAC6, the signal of Acetyl-Lys antibody recognition was weakened. The Acetyl-Lys signal is still weak in the presence of HDAC6 and 1mM inhibitor ACY-1215. However, with the increase of ACY-1215 concentration, the Acetyl-Lys signal was significantly enhanced in the presence of both HDAC6 and 2 mM inhibitor ACY-1215. These results indicate that HDAC6 can remove part of the acetylation modification of STAT1. 2 mM ACY-1215 partially restored STAT1 acetylation levels by inhibiting HDAC6.

To investigate whether inhibition of STAT1 and NF-κB p65 into the nucleus by ACY-1215 affects their transcriptional regulation of PD-L1, the experiments with luciferin reporter vector constructed by transcriptional regulatory sequences of upstream to downstream (− 1940 bp ~ + 87 bp) of the transcription start site of PD-L1 were conducted. When the transfected cells were pretreated with IFN-γ and then treated with different concentrations of ACY-1215, luciferase activity decreased as the concentration of ACY-1215 increased (Fig. 5G,H). STAT1 and NF-κB p65 are not only the important transcription factors of PD-L1, but also regulate many other important target genes. c-Myc is a downstream target gene of STAT1, and BCL-2 is a downstream target gene of both STAT1 and NF-κB. In order to understand the regulatory effects of ACY-1215 on other target genes of STAT1 and NF-κB p65, the correlation analysis using the online website TIMER was conducted. In colorectal cancer, the expression of HDAC6 was significantly positively correlated with MYC, p65, and BCL2L1 (*p* < 0.01) (Fig. S4A), while negatively correlated with infiltration levels of CD8^+^T cells (Fig. S4B). This further verified the adverse effect of high HDAC6 expression on colorectal cancer patient.

**Fig. 6:**
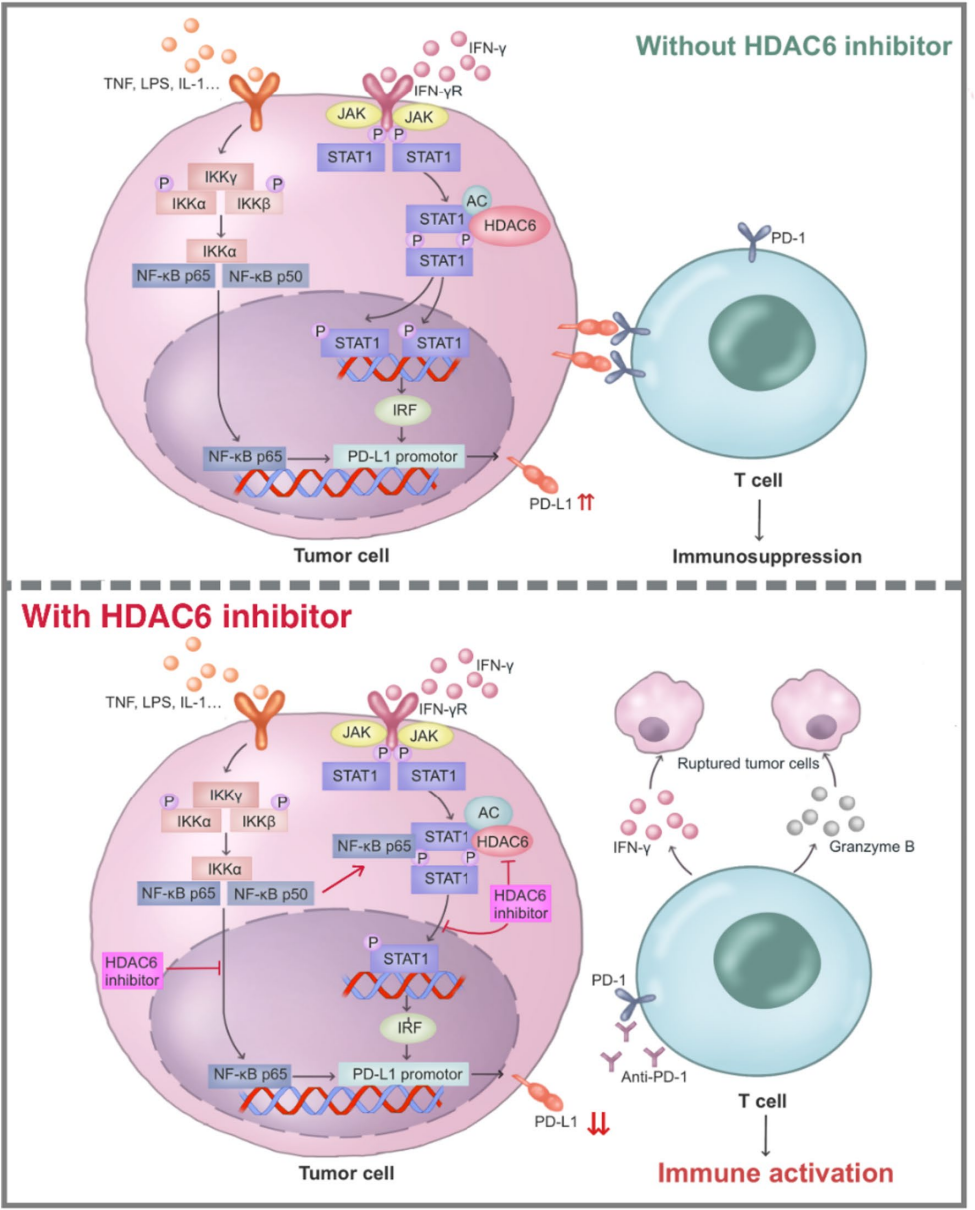
The mechanisms by which HDAC6 inhibitor (ACY-1215) down-regulates PD-L1 and promotes T cell activation.  (Upper) In the absence of HDAC6 inhibitors, the activity of HDAC6 in tumor cells is high. HDAC6 interacts with STAT1. HDAC6 deacetylates STAT1, which keeps low STAT1 acetylation. When the NF-κB and STAT1 signaling pathways are activated, NF-κB p65 and STAT1 enter the nucleus and promote the activation of downstream target genes, including PD-L1. The highly expressed PD-L1 (ligand) binds to the PD-1 (receptor) on the surface of T cells, inhibits the activity of T cells, enabling immune escape of tumor cells. (Bottom) When treating tumor cells with the HDAC6 inhibitors (ACY-1215), ACY-1215 inhibits the entry of STAT1 and NF-κB p65 into the nucleus to exert transcriptional regulation on PD-L1 by promoting the acetylation of STAT1, leading to downregulation of PD-L1 expression and thus promoting the activation of T cells

Since ACY-1215 regulated the acetylation and phosphorylation of STAT1, it’s worth speculating that STAT1 may be a non-histone substrate for HDAC6. Interactions between endogenous STAT1 and HDAC6 were observed by co-immunoprecipitation (Co-IP) experiments (Fig. 5I). In addition, immunofluorescence assay showed significant colocalization of endogenous STAT1 and HDAC6 (Fig. 5J, K). In summary (Fig. 6), our study showed that when treating tumor cells with the HDAC6 inhibitors (ACY-1215), on the one hand, ACY-1215 increased the acetylation level of STAT1 and inhibited the phosphorylation of STAT1, preventing STAT1 from entering the nucleus to play a transcriptional regulatory role in PD-L1. On the other hand, STAT1 with increased acetylation level also formed a complex with NF-κB p65, which could inhibit NF-κB p65 from entering the nucleus and prevented its transcriptional regulation of PD-L1, resulting in decreased expression level of PD-L1. In addition, ACY-1215 contributed to the T-cell killing effect on tumor cells by promoting T cell activation.

## Discussion

The immune checkpoint inhibitors have shown good therapeutic effect in a variety of tumors [37]. However, the problem of resistance and the low clinical response rate are limiting further development of the immune checkpoint inhibitors. The combined application of multiple anti-tumor therapeutic strategies has become the focus of most researchers. ACY-1215 is a potent inhibitor of HDAC6 that inhibits a variety of tumors [38–40]. Studies have shown that HDAC6 selective inhibitors have the additive effect in tumor therapy when used in combination with other anti-tumor drugs [41–46]. In this study, we report that ACY-1215 combined with anti-PD1 can effectively inhibit the growth of subcutaneous tumor in mice. This provides a new combination strategy for immunotherapy, expanding the use of the HDAC6 inhibitor in solid tumors.

Among the HDAC6 selective inhibitors that have been developed, ACY-1215 has an IC50 of 4.7 nM against HDAC6, making it the first orally highly selective HDAC6 inhibitor [16, 47]. Researchers have revealed that ACY-1215 plays an important role in the biological processes of tuppmor cells [17, 19, 48–51]. ACY-1215 also plays a regulatory role in tumor immunity. In melanoma, ACY-1215 down-regulates mTORC1/2 signaling and inhibits the production of Treg cells and Th2 cytokines [23]. ACY-1215 combined with the BRD4 inhibitor (JQ1) inhibited the growth of xenograft tumors from small cell lung cancer cells by stimulating NK cell-mediated immune activity [24]. Here, we conducted a pathological analysis of mouse tumor tissue and found that ACY-1215 may play a role in inhibiting tumor growth in colorectal cancer by promoting anti-tumor immunity. This finding shows the potential of ACY-1215 in the future research and application of tumor immunotherapy.

As a key immune checkpoint protein, PD-L1 binds to the PD-1 receptor on the surface of T cells and activates co-inhibitory signal transduction to inhibit the function of cytotoxic T lymphocytes, leading to immunosuppression and enabling tumor cells to evade the surveillance and attack of the immune system [52, 53]. The expression level of PD-L1 in various tumor cells is higher than that in normal cells, which is associated with poor prognosis of patients [54, 55]. Regarding the study on the regulation of PD-L1 expression by HDAC6, it has been reported that when HDAC6 is highly expressed in melanoma, p-STAT3 and HDAC6 bind the promoter of PD-L1, promoting the transcription of PD-L1 [21, 22]. The HDAC6 selective inhibitor designed and synthesized by Tao et al. can suppress IL-6-mediated PD-L1 expression in non-small cell lung cancer cells [33]. In the current study, the in vitro and in vivo experiments showed that ACY-1215 could down-regulate the IFN-γ-induced PD-L1 expression in colorectal cancer cells.

HDAC6 is the most widely studied class IIb HDAC, and its unique structure and cytoplasmic localization distinguish it from other members of the HDAC family [56]. In addition to regulating histones, HDAC6 can also act on many cytoplasmic non-histone proteins [57]. Both HDAC6 deletion and inhibition lead to the inhibition of α-tubulin acetylation and effectively block endotoxin-induced STAT1 activation [58]. These studies suggest that inhibition of HDAC6 exerts some regulatory effect on STAT1, but do not prove that STAT1 is the substrate regulated directly by HDAC6. In this study, we revealed that HDAC6 interacts and co-localizes with STAT1 endogenously, indicating that STAT1 can be regulated by HDAC6 as a substrate molecule. In this case, does ACY-1215, a selective HDAC6 inhibitor, regulate the acetylation level of STAT1? Previous studies have shown that the acetylation of STAT1 can inhibit IFN-α-induced STAT1 phosphorylation [34], suggesting some relationship between the acetylation and phosphorylation modulation. We found that when ACY-1215 inhibited the activity of HDAC6, it increased the acetylation level of STAT1, while the phosphorylation of STAT1 was inhibited. IFN-γ-activated STAT1 signaling pathway is the main cause of PD-L1 up-regulation. We demonstrated that ACY-1215 blocked STAT1 nuclear translocation, which in turn prevented STAT1 transcriptional regulation of PD-L1. In addition, STAT1 with elevated acetylation levels can also form complexes with NF-κB p65, thereby inhibiting the nucleation of NF-κB p65 and preventing its transcriptional regulation of PD-L1. This further enriches the regulatory mechanism of PD-L1 expression. Using the online database, we also found that HDAC6 was positively correlated with the downstream target genes of STAT1 or NF-κB p65, such as MYC, RELA (p65) and BCL2L1, in colorectal cancer specimens. These findings suggest that HDAC6 plays a crucial role in the development of tumors, and may become a key target of tumors. At the same time, it provides a basis for the further application and development of HDAC6 inhibitors in solid tumors.

It has been shown that HDAC6 promotes the occurrence and development of tumors by affecting tumor immunogenicity and immune cell activity. HDAC6 inhibitors can inhibit the function of tumor-associated macrophages and myeloid-derived suppressor cells in the tumor microenvironment, and promote the activity of cytotoxic T cells and memory T cells and their killing effect on tumor cells [59]. HDAC6 inhibitors can also transform Treg cells and M2 macrophages, thereby increasing the number of cytotoxic T cells and M1 macrophages to reshape the tumor immune microenvironment [60–62]. We analyzed the correlation between HDAC6 expression and immune cell infiltration in colorectal cancer and found that HDAC6 expression was significantly negatively correlated with the infiltration level of CD8^+^T cells. These suggest that HDAC6 inhibitors may be of great significance in the study of tumor immunotherapy.

## Conclusions

In summary, our study showed that the HDAC6 inhibitor (ACY-1215) inhibits the entry of STAT1 and NF-κB p65 into the nucleus to exert transcriptional regulation on PD-L1 by promoting the acetylation of STAT1, leading to downregulation of PD-L1 expression and thus promoting the activation of T cells (Fig. 6). This study reveals a novel regulatory mechanism of HDAC6 on non-histone substrates, especially on protein acetylation. HDAC6 inhibitors may be of great significance in the study of tumor immunotherapy and related combination strategies, which provides a new strategy for tumor immunotherapy of colorectal cancer.

### Electronic supplementary material

Below is the link to the electronic supplementary material.

Supplementary Material 1 (DOCX 1834 kb)

Supplementary Material 2 (DOCX 29 kb)

## Data Availability

The data presented in this study are available on request from the corresponding author.
