# Supplementary material for: HDAC6 inhibitor ACY-1215 enhances STAT1 acetylation to block PD-L1 for colorectal cancer immunotherapy
Source: Cancer Immunol Immunother. 2024 Jan 17;73(1):7. doi: 10.1007/s00262-023-03624-y (PMC10794344; doi:10.1007/s00262-023-03624-y)
Supplement: Supplementary file 1 — Supplementary Material 1 (DOCX 1834 kb) [file 262_2023_3624_MOESM1_ESM.docx]

Supplementary
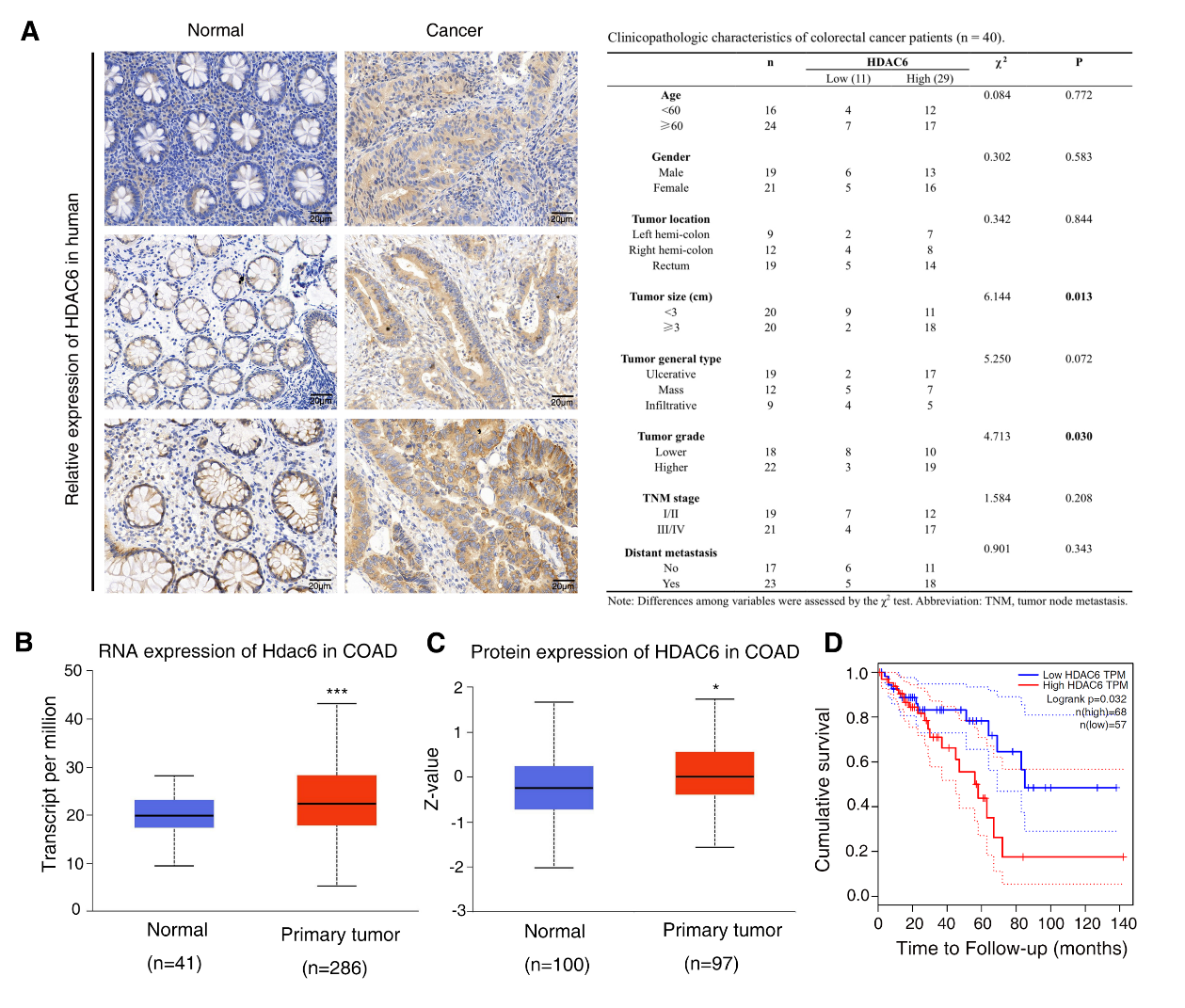
Figure 1. HDAC6 is highly expressed in colorectal cancer.

(A) The expression of HDAC6 in colorectal cancer tissues and the adjacent tissues of patients were assayed by immunohistochemistry. Scale bars: 20 μm.

(B-C) The expression of HDAC6 in colorectal cancer tissues or normal tissues in TCGA (B) and CPTAC (C) databases was analyzed through the website of UALCAN (http://ualcan.path.uab.edu/).

(D) The association between the expression of HDAC6 and survival of the colorectal cancer patients in the TCGA database was analyzed through the website of GEPIA (http://gepia.cancer-pku.cn/).


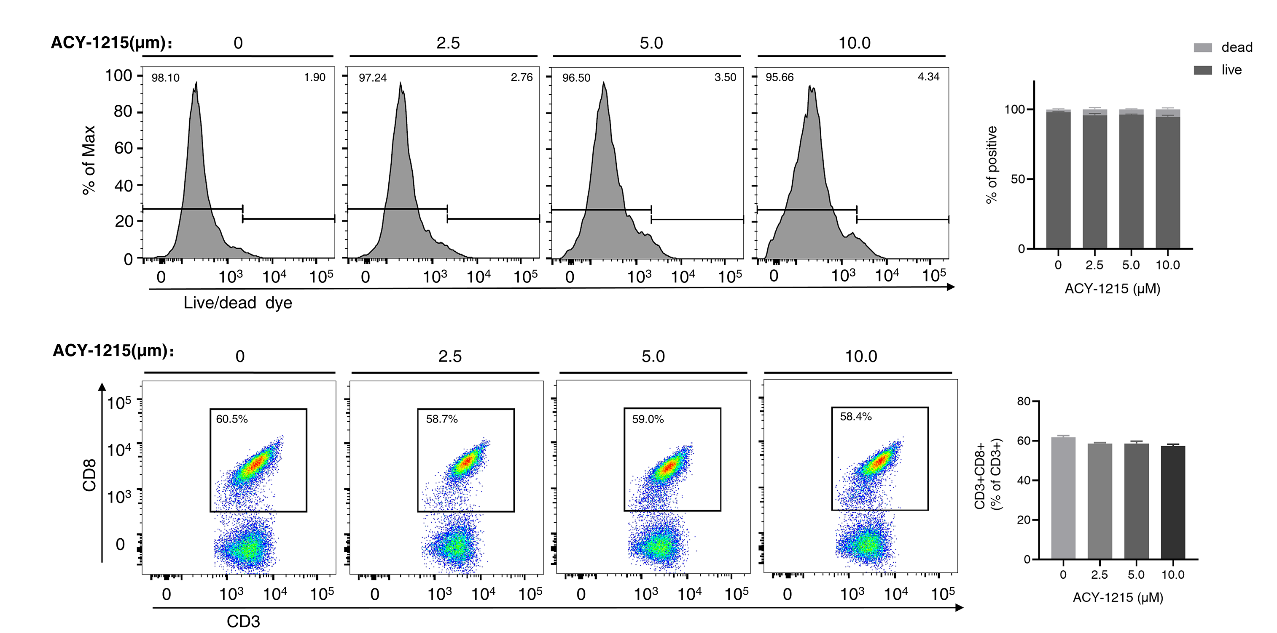


Supplementary Figure 2. ACY-1215 treatment had no effect on the activity of CD3^+^ T cells and the number of CD8^+^ T cells.

SW480 cells were treated with IFN-γ for 24 h, then incubated with ACY-1215 (0, 2.5, 5.0, 10.0 µM) and activated T cells for 24 h. The ratio of SW480 cells to T cells was 1:8. After treatment, T cells suspended in the medium in the co-culture system were collected as far as possible. The effects of ACY-1215 (0, 2.5, 5.0, 10.0 µM) on the total T cells (CD3^+^) activity and cytotoxic T cell (CD8^+^) in total T cells was assayed by flow cytometry.


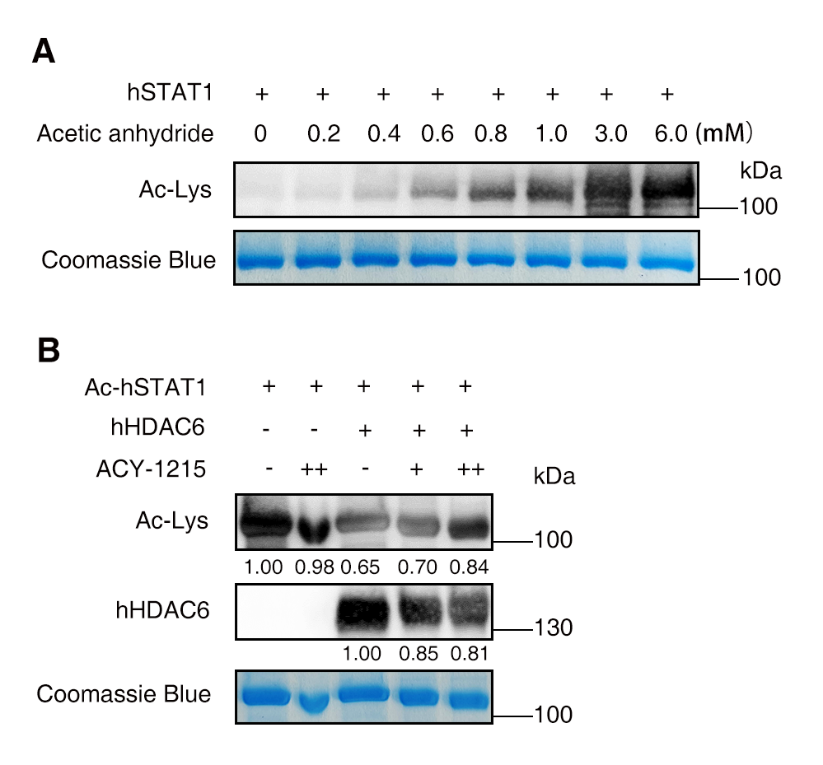
Supplementary Figure 3. HDAC6 mediates the deacetylation of STAT1 protein in vitro.

(A) Western blot analysis of lysine acetylation modification of 1 μg STAT1 in acetic anhydride solution with different concentrations. Acetyl-Lys antibody was used to detect the acetylation modification at lysine sites, and Coomassie blue staining was used to reflect the total STAT1 protein in each reaction system.

(B) Western blot analysis of lysine acetylation modification of acetylated STAT1 in the HDAC6 deacetylation reaction system. ACY-1215 is an inhibitor of HDAC6 deacetylase activity ("+" means the final concentration of ACY-1215 is 1mM; "++" means the final concentration of ACY-1215 is 2mM). Acetyl-Lys antibody was used to detect the acetylation modification at the lysine site, HDAC6 antibody was used to detect the addition of HDAC6 to the reaction, and Coomassie blue staining reflected the total STAT1 protein (including acetylation and deacetylation) of each reaction system.


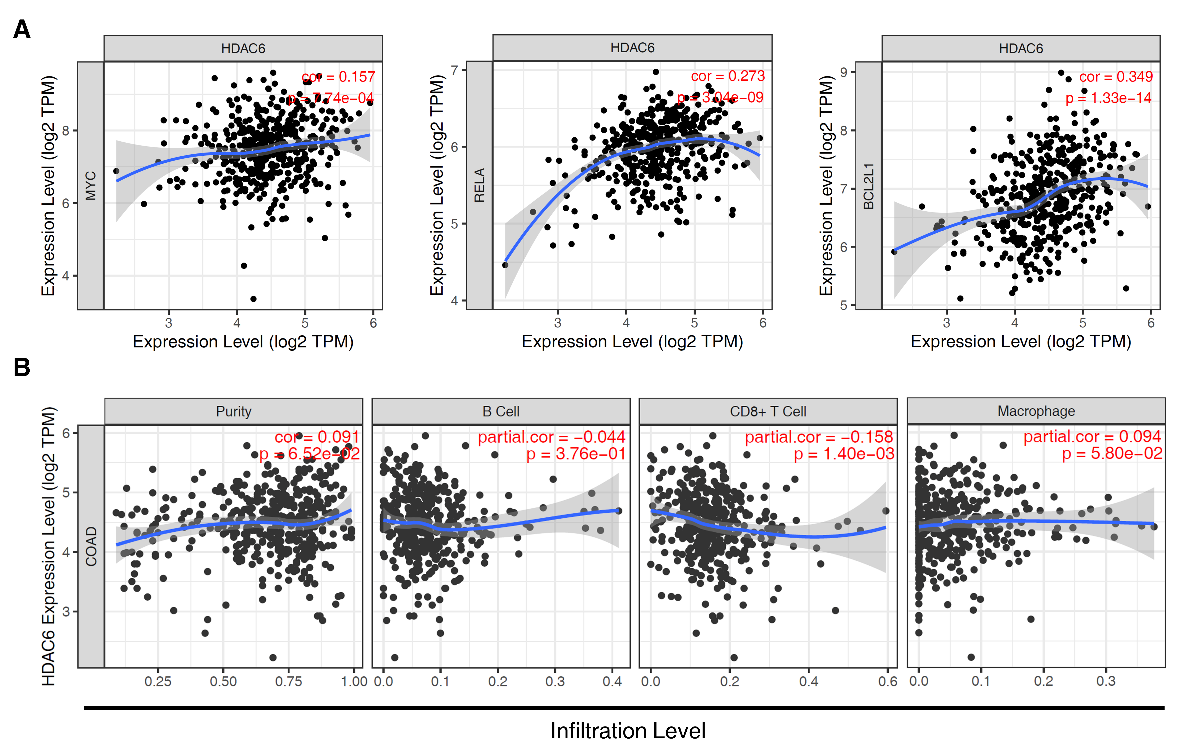


Supplementary Figure 4. The correlation analysis of HDAC6 expression with the downstream target genes of STAT1 or NF-kB, and with the infiltration level of immune cells.

The correlation analysis was performed via the website of TIMER (<https://cistrome.shinyapps.io/timer/>).

(A) The correlation between HDAC6 expression and the expression of MYC, RELA (p65), or BCL2L1 in colorectal cancer.

(B) The correlation between HDAC6 expression and the infiltration level of immune cells in colorectal cancer.
