# Supplementary material for: HDAC6 inhibitor ACY-1215 enhances STAT1 acetylation to block PD-L1 for colorectal cancer immunotherapy
Source: Cancer Immunol Immunother. 2024 Jan 17;73(1):7. doi: 10.1007/s00262-023-03624-y (PMC10794344; doi:10.1007/s00262-023-03624-y)
Supplement: Supplementary file 2 — Supplementary Material 2 (DOCX 29 kb) [file 262_2023_3624_MOESM2_ESM.docx]

**Supplemental Table 1. The primers for quantitative PCR.**

| Gene name | Sequence（5’ to 3’） |
| --- | --- |
| Mouse *Pd-l1* | Forward：GTCAATGCCCCATACCGCAA |
|  | Reverse：GGCCTGACATATTAGTTCATGCT |
| Human *PD-L1* | Forward：GGTGCCGACTACAAGCGAAT |
|  | Reverse：TGGTGGTGGTGGTCTTACC |
| Mouse *Ifn-γ* | Forward：ATGAACGCTACACACTGCATC |
|  | Reverse：CCATCCTTTTGCCAGTTCCTC |
| Mouse *Gapdh* | Forward：TCTGACGTGCCGCCTGGAGA |
|  | Reverse：CAGCCCCGGCATCGAAGGTG |
| Human *GAPDH* | Forward：AACGGATTTGGTCGTATTGG |
|  | Reverse：TTGATTTTGGAGGGATCTCG |

**Supplemental Table 2. Key products .**

| **Reagen or resource** | **Source** | | **Identifier** |
| --- | --- | --- | --- |
| **Inhibitor/antibody drugs** |  |  | |
| HDAC6 inhibitor ACY-1215 | Selleck | Cat# S8001 | |
| Anti mouse PD-1 | BioXCell | Cat# BP0146 | |
| **Antibody** |  |  | |
| PD-L1/CD274 Monoclonal Antibody | Proteintech | Cat# 66248-1-Ig | |
| Rabbit Anti-IFN gamma antibody | Bioss | Cat# bs-0480R | |
| Anti-GAPDH Recombinant Rabbit Monoclonal Antibody | HUABIO | Cat# ET1601-4 | |
| 重组Anti-HDAC6抗体 | Abcam | Cat# ab133493 | |
| CD8A Rabbit pAb | ABclonal | Cat# A11856 | |
| TBX21, T-bet Polyclonal antibody | Proteintech | Cat# 13700-1-AP | |
| STAT1 Polyclonal antibody | Proteintech | Cat# 10144-2-AP | |
| Rabbit monoclonal to Granzyme B | Abcam | Cat# ab255598 | |
| Phospho-STAT1 (Tyr701) Antibody | Affinity | Cat# AF3300 | |
| Anti-NF-κB p65 Recombinant Rabbit Monoclonal Antibody | HUABIO | Cat# ET1603-12 | |
| Histone H3 Rabbit pAb | ABclonal | Cat# A2348 | |
| Pan Acetyl-Lysine Rabbit pAb | ABclonal | Cat# A2391 | |
| Goat anti-Mouse IgG (H+L) Secondary Antibody, HRP | Abiowell | Cat# AWS0001a | |
| Goat anti-Rabbit IgG (H+L) Secondary Antibody, HRP | Abiowell | Cat# AWS0002a | |
| IFluor594 Conjugated Goat anti-mouse IgG Goat Polyclonal Antibody | HUABIO | Cat# HA1126 | |
| Mouse IgG | HUABIO | Cat# HA1027 | |
| Rabbit IgG | HUABIO | Cat# HA1002 | |
| PE anti-human CD274 (PD-L1) Antibody | BioLegend | Cat# 329706 | |
| APC/Cvanine7 anti-human CD3 | BioLegend | Cat# 300317 | |
| PE/Cyanine7 anti-human CD8a | BioLegend | Cat# 300913 | |
| Brilliant Violet 421^TM^ anti-human IFN-γ | BioLegend | Cat# 502531 | |
| PE anti-human/mouse Granzyme B Recombinant | BioLegend | Cat# 372207 | |
| **Strains and plasmid vectors** |  |  | |
| DH5α Chemically Competent Cell | Tsingke Biotechnology | Cat# TSC-C14 | |
| Human STAT1 cDNA Clones | Sino Biological Inc. | Cat# HG12766-UT | |
| Human RELA/NF-kappa B p65 cDNA Clones | Sino Biological Inc. | Cat# HG12054-UT | |
| **Recombinant protein** |  |  | |
| STAT1 Protein, Human, Recombinant | Sino Biological Inc. | Cat# 12766-H20B | |
| Recombinant HDAC6 protein | Active Motif | Cat# 31543 | |

| **Continued** |  | |  |
| --- | --- | --- | --- |
| **Reagen or resource** | **Source** | | **Identifier** |
| **Reagent/Kit** |  |  | |
| Stripping Buffer | Jiangsu Cowin Biotech | Cat# CW0056M | |
| RIPA Lysis Buffer（Strong） | Jiangsu Cowin Biotech | Cat# CW2333S | |
| Protease Inhibitor Cocktail | Bimake | Cat# B14001 | |
| Phosphatase Inhibitor Cocktail | Bimake | Cat# B15001 | |
| 180 kDa Prestained Protein Marker | Vazyme Biotech | Cat# MP102-01 | |
| PMSF | Beyotime Biotechnology | Cat# ST506 | |
| NCM Universal Antibody Diluent | NCM Biotech | Cat# WB100D | |
| 6×Protein Loading Buffer | TransGen Biotech | Cat# DL101-02 | |
| DAPI solution (Nuclear Labeling) | Bioss | Cat# C02-04002 | |
| Mounting Medium, antifading | Solarbio | Cat# S2100 | |
| Eosin | Abiowell | Cat# AWI0029a | |
| Mayer Hematoxylin solution | Abiowell | Cat# AWI0009a | |
| Citrate Antigen Retrieval Solution | Jiangsu Cowin Biotech | Cat# CW0128S | |
| Anticoagulant for cell separation | TBD | Cat# TBDTM-0200 | |
| Immuno Cult Human CD3/CD28/CD2 T cell activator | Stemcell | Cat# 10970 | |
| Human peripheral blood lymphocyte isolation fluid | TBD | Cat# LTS1077 | |
| Protein A/G magnetic bead | Biolinkedin | Cat# L-1004 | |
| Brefeldin | MedChemExpress | Cat# HY-16592 | |
| Recombinant Human IL-4 | PeproTech | Cat# 200-04 | |
| Recombinant Human GM-CSF | PeproTech | Cat# 300-03 | |
| Recombinant Human IL-7 | PeproTech | Cat# 200-07 | |
| Recombinant Human IL-15 | PeproTech | Cat# 200-15 | |
| Interferon Gamma Protein, Human, Recombinant | Sino Biological Inc. | Cat# 11725-HNAS | |
| Murine IFN-gamma | PeproTech | Cat# 315-05-20 | |
| Recombinant Human IL-2 | PeproTech | Cat# 200-02-50 | |
| 30% Polyacrylamide solution | Biosharp | Cat# BL513B | |
| TEMED | Jiangsu Cowin Biotech | Cat# 10335 | |
| SDS | Sigma | Cat# STBJ9261 | |
| DMSO | MP biomedicals | Cat# 196055 | |
| Tween-20 | Dingguo | Cat# DH358-4 | |
| QmSuero® Fetal Bovine Serum | Tsingmu Biotechnology | Cat# mu001SR | |
| Trizol | MRCgene | Cat# TR118 | |
| RPMI-1640 medium | BasalMedia | Cat# M211017 | |
| DMEM, high glucose, with glutamine | Biological Industries | Cat# 01-052-1A | |
| Penicillin-Streptomycin Solution | Pricella | Cat# PB180120 | |
| Trypsin EDTA Solution | Biological Industries | Cat# 03-050-1A | |
| **Continued** |  |  | |
| **Reagen or resource** | **Source** | **Identifier** | |
| **Reagent/Kit** |  |  | |
| Lipofectamine3000 | Invitrogene Thermo | Cat# 2410770 | |
| 2x SYBR Green qPCR Master Mix | Bimake | Cat# B21202 | |
| Absolute ethyl alcohol | Sinopharm Group | Cat# 10009218 | |
| Trichloromethane | Sinopharm Group | Cat# 10006818 | |
| Isopropanol | HengXing Chemical Reagent | Cat# 32064 | |
| Xylol | Sinopharm Group | Cat# 10023418 | |
| Rhamsan gum | Shanghai Yiyang Industrial | Cat# 20200912 | |
| Acetic anhydride | Chengdu Kelong chemical reagent factory | Cat# 05.001.0504 | |
| Coomassie Brilliant Blue quick dye solution | Shanghai Epizyme Biomedical Technology | Cat# PS111 | |
| Glycine | Sigma | Cat# WXBD5703V | |
| Trisma base | Sigma | Cat# WXBC7884V | |
| Methyl alcohol | HengXing Chemical Reagent | Cat# 32058 | |
| Triton-X | BBIFIL | Cat# 9002-93-1 | |
| Micro BCA Protein Assay Kit | Jiangsu Cowin Biotech | Cat# CW0014S | |
| RevertAid First Strand cDNA Synthesis Kit | Thermo | Cat# K1622 | |
| EndoFree Mini Plasmid Kit | TIANGEN | Cat# DP118-02 | |
| Cell Counting Kit-8 (CCK-8) | AOExBIO | Cat# K1018 | |
| Nuclear and Cytoplasmic Protein Extraction Kit | Beyotime Biotechnology | Cat# P0027 | |
| TransDetect Double-Luciferase Reporter Assay Kit | TransGen Biotech | Cat# FR201-01 | |
|  |  |  | |
